# Potential of essential oils for protection of grains contaminated by aflatoxin produced by *Aspergillus flavus*

**DOI:** 10.3389/fmicb.2014.00269

**Published:** 2014-06-04

**Authors:** Renata H. Esper, Edlayne Gonçalez, Marcia O. M. Marques, Roberto C. Felicio, Joana D. Felicio

**Affiliations:** ^1^Centro de Pesquisa e Desenvolvimento em Sanidade Animal, Instituto BiológicoSão Paulo, Brazil; ^2^Centro de Pesquisa e Desenvolvimento de Recursos Genéticos Vegetais, Instituto AgronômicoCampinas, Brazil; ^3^Departamento de Ciências Exatas e Tecnológicas, Universidade Estadual de Santa CruzIlhéus, Brazil

**Keywords:** corn, soybeans, essential oil, mycotoxins, *Origanum vulgare*, *Ageratum conyzoides*

## Abstract

Aflatoxin B_1_ (AFB_1_) is a highly toxic and carcinogenic metabolite produced by *Aspergillus* species on food and agricultural commodities. Inhibitory effects of essential oils of *Ageratum conyzoides* (mentrasto) and *Origanum vulgare* (oregano) on the mycelial growth and aflatoxin B_1_ production by *Aspergillus flavus* have been studied previously in culture medium. The aim of this study was to evaluate aflatoxin B_1_ production by *Aspergillus flavus* in real food systems (corn and soybean) treated with *Ageratum conyzoides* (mentrasto) and *Origanum vulgare* (oregano) essential oils. Samples with 60 g of the grains were treated with different volumes of essential oils, 200, 100, 50, and 10 μL for oregano and 50, 30, 15, and 10 μL for mentrasto. Fungal growth was evaluated by disk diffusion method. Aflatoxin B_1_ production was evaluated inoculating suspensions of *A. flavus* containing 1.3 × 10^5^ spores/mL in 60 g of grains (corn and soybeans) after adjusting the water activity at 0.94. Aflatoxin was quantified by photodensitometry. Fungal growth and aflatoxin production were inhibited by essential oils, but the mentrasto oil was more effective in soybeans than that of oregano. On the other hand, in corn samples, the oregano essential oil was more effective than that of mentrasto. Chemical compositions of the essential oils were also investigated. The GC/MS oils analysis showed that the main component of mentrasto essential oil is precocene I and of the main component of oregano essential oil is 4-terpineol. The results indicate that both essential oils can become an alternative for the control of aflatoxins in corn and soybeans.

## Introduction

Loss of food commodities due to pest infestations is a major reason of food crisis particularly in tropical countries (Prakash et al., 2013). Grains and cereals are exposed in the field and also during the storage subjected to a wide variety of organisms. Among these organisms, fungi can cause a lot of damage to the grains during planting, harvesting, and storage (Sillker and Elliot, 1980). The economic impact of fungal invasion include the decline of the power of germination, moldering visible discoloration, odor, chemical, and nutritional changes, with consequent loss of quality, mycotoxin production, making them unfit for consumption (Paster and Bullerman, 1988). The grains serve as a suitable substrate for mold growth and mycotoxin production (Bryden, 2012). The presence of molds and mycotoxin in food commodities is a potential health threat to humans and livestock.

Mycotoxins are toxic substances that can enter the human and animal food chain, not only by the direct ingestion of contaminated seeds or processed food, but also by the consumption of meat or other animal products (i.e., milk) from livestock fed with feed and contaminated silages (Zjalic et al., 2006).

Aflatoxins are mycotoxins produced especially by *Aspergillus flavus, A. parasiticus*, and *A. nomius*, and they are mutagenic and carcinogenic in animals and humans. Overall, there are a number of approaches that can be taken to minimize mycotoxin contamination in animal feeds and these involve prevention of fungal growth and therefor mycotoxin formation and strategies to reduce or eliminate mycotoxins from contaminated commodities, especially feed additives (Bryden, 2012). The exploitation of natural substances with bioactivity against fungi has been the target of interest in the search for ecologically safe products (Passone et al., 2012).

Of many approaches investigated to manage aflatoxin contamination, chemical control methods using essential oils have shown great promise. Numerous essential oils produced by medicinal plants have been tested for their ability in controlling aflatoxin contamination in culture medium conditions (Omidbeygi et al., 2009; Kumar et al., 2010; Nogueira et al., 2010; Medeiros et al., 2011), but there is scanty information concerning essential oils for protection of grains contaminated with aflatoxins. In recent studies, the literature relates antifungal and antiaflatoxigenic activity by essential oils on peanut, wheat, and chickpea (Passone et al., 2013; Kedia et al., 2014).

*Ageratum conyzoides* (mentrasto) and *Origanum vulgare* (oregano) essential oils have been reported as inhibitors of growth and production of aflatoxin B_1_ by *A. flavus* in culture medium (Montes-Belmont and Carvajal, 1998; Nogueira et al., 2010), but in real food systems (corn and soybean) no reports. In this work corn and soybean grains, experimentally contaminated with *A. flavus*, were treated with *A. conyzoides* and *O. vulgare* essential oils to evaluate aflatoxin B_1_ production. The chemical compositions of both essential oils also were investigated.

## Materials and methods

### Plant material

Aerial parts of *A. conyzoides* were collected in Ibiúna City, São Paulo State, Brazil. A voucher specimen (N° PMSP 9686b) was deposited in the herbarium of the City Hall of São Paulo City. *O. vulgare* was bought in shops in the city of São Paulo, Brazil.

### Oil extraction and analysis

Fresh leaves of *A. conyzoides* cut into small pieces and dried *O. vulgare* were placed in a distillation Clevenger apparatus for 2 h. The hydrolytes were extracted with hexane and evaporated at room temperature, and the resulting oil was stored in dark glass bottles in a freezer until be used in *A. flavus* and GC/MS analysis.

GC/MS analyses of the main components of the essential oils were performed on a Shimadzu QP-5000 equipped with an OV-5 (30 mm × 0.25 mm × 0.25 μm, Ohio Valley Specialty Chemical, Inc.) capillary column. Operating conditions were undertaken at oven temperature from 60 to 240°C at 3°C min^−1^, injector and detector temperatures of 240 and 230°C, respectively, at 70 eV. Helium was used as a carrier gas at a constant flow of 1.7 mL min^−1^, split 1/20. The oil components were identified using retention indices with those of authentic compounds or with literature data (McLafferty and Stauffer, 1989; Adams, 2001).

### Culture conditions

The aflatoxin B1 producing strain of *A. flavus* was isolated from soil of a crop rice. It was assigned by the Reference Laboratory of Microbiology located at the Instituto de Tecnologia de Alimentos (ITAL), Campinas City, Brazil. The strain was kept lyophilized in freezer until ready to use.

The strain was reconstituted in sterile water and inoculated in potato dextrose agar (Difco Laboratories) for 10 days at 25°C. After this period, the cultures are washed with a sterile solution of 1 % Tween 80.

### Test of antifungal activity of essential oils

#### Disk diffusion assay

Filter paper disks (6 mm diameter) containing 5.0 μL of essential oils of *A. conyzoides* and *O. vulgare* were applied on the surface of potato dextrose agar (Difco Laboratories) plates previously inoculated with *A. flavus*. The inoculated plates were incubated at 25°C for 5 days. At the end of the period, antifungal activity was evaluated by measuring the zone of inhibition (mm) against the test fungus (Yin and Tsao, 1999).

#### Test in grains (in vivo)

The fungal growth was also evaluated in grains, using the same methodology above, but the paper disc was replaced by grains (corn and soybeans).

Commercial fungicide was used as a positive control. All treatments consisted of three replicates and repeated once more. The averages of the experimental results were determined.

### Evaluation of aflatoxin production in corn and soybeans impregnated with essential oils

Samples with 60 g of each grain (corn and soybeans) were irradiated with a dose of 20 kGy, at the Instituto de Pesquisas Energéticas Nucleares (São Paulo City), to eliminate the fungal contamination. For evaluation of aflatoxin B1 production in the grains, the samples were placed in erlenmeyer flasks; the water activity was adjusted to 0.94, and the samples were treated with 200, 100, 50, and 10 μL of oregano essential oil and 50, 30, 15, and 10 μL of mentrasto essential oil. Untreated samples were used as control.

Suspensions of *A. flavus* containing 1.3 × 10^5^ spores/mL were inoculated in the grains treated and untreated with the essential oils. Four replicates were performed for each treatment, and the experiment was repeated twice.

The cultures were incubated at 25°C for 10 days for aflatoxin production, then grain samples were ground. Thereafter, 50 g of each sample was used for extraction of aflatoxin through the method of Soares and Rodriguez-Amaya (1989). Aflatoxin B_1_ was extracted with chloroform and the solvent evaporated until 1.0 mL in a volumetric flask. An aliquot (40 μL) of each sample was spotted on silica gel-G thin layer plate (Merck, Germany) and then developed with chloroform:acetone 9:1 (v:v) as a solvent system. The concentration of aflatoxin B_1_ was determined by photodensitometry (Shimadzu, CS 9000) comparing the area of the spot samples with aflatoxin B_1_ standards (Sigma Aldrich, USA). The quantification and detection limits for AFB_1_ were 0.08 and 0.04 ng, respectively.

### Statistical analysis

The experiments were analyzed by ANOVA and Tukey's multiple range tests with a significance level *P* < 0.05. The IC_50_ was calculated by GraphPad Prim 5.0 software.

## Results

### Chemical composition

The essential oils extracted from *A. conyzoides* and *O. vulgare* yielded 0.042 and 0.27%, respectively. Chemical analysis by GC/MS of the components of the oils led to identification of 5 and 18 components of the essential oil of *A. conyzoides* and of that of *O. vulgare*, respectively (Tables 1, 2). The major components of *A. conyzoides* oil were precocene I (96.53%) and precocene II (2.40%) and those of *O. vulgare* essential oil were 4-terpeniol (44.11%), linalool (15.22%), and thymol (13.40%).

**Table 1:** **Components identified as constituents of essential oil of *Ageratum conyzoides* (%)**.

**Constituents**	***RT* (min.)**	**[%]**	IR^*^	IR^**^
n-Tetradecane	23.540	tr	1400	1400
Trans-caryophillene	24.198	tr	1416	1418
α-Humulene	25.562	tr	1449	1454
Dimetoxy ageratocromene (Precocene I)	25.749	96.53	1454	1463
Ageratocromene (Precocene II)	33.220	2.40	1659	1660

* Experiment retention indices;

** literature retention indices (Adams, 2001); RT, retention time; tr, traces of substance (tr ≤ 0.59).

**Table 2:** **Components identified as constituents of essential oil of *Oreganum vulgare* (%)**.

**Constituents**	***RT* (min.)**	**[%]**	IR^*^	IR^**^
o-Cimene	7.974	0.99	1013	1022
p-Cimene	8.194	1.08	1019	1026
Limonene	8.388	0.26	1025	1031
γ-Terpinene	9.421	5.02	1053	1062
Trans-sabinene hidrate	9.688	1.79	1061	–
Terpinolene	10.520	0.70	1084	1088
Linalool	10.827	15.22	1093	1098
Trans-p-mentenol	10.892	3.31	1095	–
Cis-p-mentenol	11.732	1.99	1116	–
Borneol	12.433	1.34	1133	–
4-terpineol	13.981	44.11	1171	1177
α-Terpineol	14.493	5.96	1184	1189
Dihydro carveol	14.733	0.36	1190	1192
2-Isopropyl-5-methyl-anisole	16.348	0.47	1228	1235
2-Isopropyl-5-methyl-anisole	16.738	1.49	1238	1244
Geraniol	17.293	1.46	1251	–

* Experiment retention indices;

** literature retention indices (Adams, 2001); RT, retention time; tr, traces of substance (tr ≤ 0.59).

### Fungal growth

The fungal growth inhibition assessed by the disk diffusion test has been used in the evaluation of plant extracts and essential oils (Reddy et al., 2008; Humeera et al., 2013).

The influence of the essential oils on the inhibitory zone against *A. flavus* were measured at 1.9 and 0.88 mm (average *n* = 6) for *A. conyzoides* and *O. vulgare*, respectively. The commercial fungicide (control) was measured at 2.15 mm. The results obtained by the disk diffusion method were 88.37 and 40.93% of inhibition of fungal growth for the *A. conyzoides* and *O. vulgare* essential oils respectively, when compared with control.

The test in grains of corn for *A. conyzoides* inhibited at 79.53% the fungal growth. But in the other experiments with grains, no halo formation around the grain, but the fungus did not grow on the grain.

### Aflatoxin production

**Figure 1:**
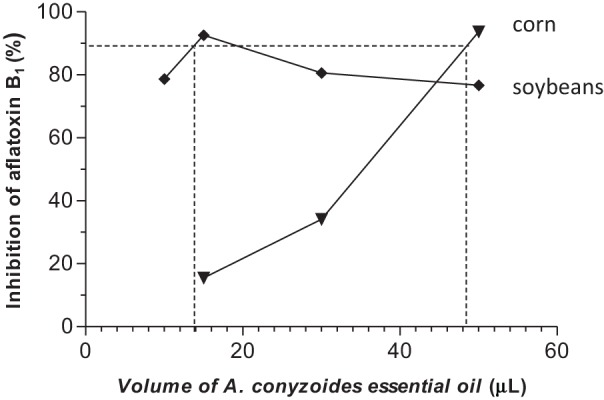
**Inhibitory effect of aflatoxin B_1_ production by different concentrations of *A. conyzoides* essential oil in corn and soybeans**.

**Figure 2:**
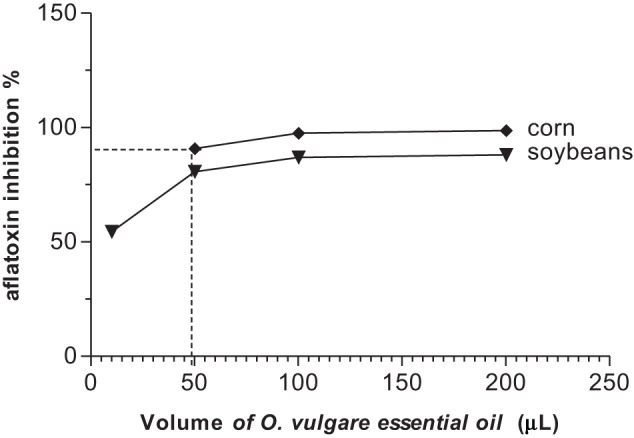
**Inhibitory effect of aflatoxin B_1_ production by different concentrations of *O. vulgare* essential oil in corn and soybeans**.

All concentrations of the two essential oils reduced aflatoxin B_1_ production in corn and soybeans samples when compared with the control (Figures 1, 2), using the same inoculum (1.3 × 10^5^ spores/mL).

The volumes 50, 30, and 15 μL of *A. conyzoides* essential oil inhibited aflatoxin B_1_ production in corn at 93.70, 34.15, and 15.45%, respectively (*P* < 0.01). The volumes 50, 30, 15, and 10 μL of the *A. conyzoides* essential oil showed inhibitory effect on aflatoxin production in soybeans above 75% when compared with the control and between their treatments (*P* < 0.001) (Figure 1).

As regards *O. vulgare* essential oil, the volumes of 10, 50, 100, and 200 μL showed inhibitory effect on aflatoxin production in soybeans at 54.4, 88.68, 86.94, and 88.16%, respectively (Figure 2). There was a significant inhibition of aflatoxin B_1_ production in soybeans as compared with that of the control (*P* < 0.01). On the order hand, *O. vulgare* essential oil in corn showed an inhibitory effect up to 90% on mycotoxin/aflatoxin B1 production for all concentrations tested (*P* < 0.001) (Figure 2).

The volumes of the *A. conyzoides* essential oil that inhibited 90% of aflatoxin production were14.1 and 48.5 μL for soybeans and corn, respectively. To *O. vulgare* essential oil was 49.8 μL for corn, but was not observed for soybeans.

## Discussion

Both essential oils have been described as inhibitors of the fungal growth of *A. flavus*. Montes-Belmont and Carvajal (1998) related inhibition of *A. flavus* growth at concentration up to 1000 μg g^−1^ of *O. vulgare* essential oil. Montes-Belmont and Carvajal (1998) related that inhibition of *A. flavus* growth that occurred at concentration up to 1000 μg g^−1^ of *O. vulgare* essential oil. Nogueira et al. (2010) reported that *A. conyzoides* essential oil inhibited the fungal growth to 48% in culture medium.

The results of inhibition of fungal growth obtained allowed continuing our study, thus enabling us to verify the activity of these two essential oils in grains (corn and soybean) in order to envision their potential use as antifungal and/or antiaflatoxigenic.

The two essential oils reduced the production of aflatoxin B_1_ in corn and soybean samples when compared (Figures 1, 2). *O. vulgare* essential oil showed an inhibitory effect up to 85% in corn and soybeans at 200, 100, and 50 μL (*P* < 0.01). In the volumes of 200,100, and 50 μL of the *O. vulgare* essential oil showed significant inhibition of the aflatoxin B_1_ production in corn as compared to control (*p* < 0.001). However, in sample with 200 and 100 μL of *O. vulgare* essential oil statistically significant differences were not observed in the aflatoxin production (*p* > 0.05). The 100 μL of *O. vulgare* essential oil showed significant inhibition of aflatoxin B_1_ production in soybeans when compared to the control and among the samples (*P* < 0.01).

The statistic results and volumes of the essential oils that inhibited 90% of aflatoxin production showed that *A. conyzoides* essential oil was more active than that of *O. vulgare* in soybeans. On the other hand, in corn, the essential oil of *O. vulgare* was more active than that of *A. conyzoides*.

The results are quite interesting and corroborate with data reported by other authors that discussed activity of essential oils in real food systems.

Bluma and Etcheverry (2008) also observed a total inhibition of aflatoxin B_1_ in corn with *O. vulgare* essential oil at concentrations of 500 and 700 μg g^−1^, using a water activity of 0.955. Our results were more promising since 3.33 μg g^−1^ inhibited aflatoxin production in corn to 98.68%.

Passone et al. (2013) related that boldo (*Peumus boldus* Mol.) essential oil showed antifungal activity in peanut extract medium at doses of 1500 μL/L, that is, a highly significant effect on Aspergillus section Flavi growth rate (93 and 100% of inhibition) and aflatoxin B1 (AFB1) accumulation (100% of inhibition).

Velázquez-Nuñez et al. (2013) compared the antifungal activity efficacy of orange peel essential oil at selected concentrations, applied either by vapor exposure or direct addition on the growth of *A. flavus*. The results encompass both studied methods. *A. flavus* growth decreased as essential oil concentration increased. Although the effect of orange peel essential oil by direct addition was faster, orange peel essential oil vapors were more effective since lower concentrations of the latter were required to achieve the same antifungal effect.

Differences in the chemical composition and in the biological activity of the essential oils have been discussed by different authors. *A. conyzoides* essential oil for this study was collected in 2009, and precocene I (96.53 %) was the major constituent. Nogueira et al. (2010) collected *A. conyzoides* in 2007, at the same venue, but related that precocene II (46.35%), precocene I (42.78%) were main components. Furthermore, *A. conyzoides* essential oil collected in 2009 showed other differences in chemical makeup as: it contains fewer chemical constituents, and *n*-tetradecane is a new compound. It has not been previously reported by Nogueira et al. (2010). The differences in chemical composition of essential oils from plants could be explained due to environmental differences that occur from one year to another where they grow. Variations of oil composition of *A. conyzoides* were related by Castro et al. (2004).

Precocenes I and II have been described as inhibitors of mycotoxins' production. Yaguchi et al. (2009) related that precocenes I and II inhibited 3-acetyldeoxynivalenol production by *Fusarium graminearum* in a liquid culture with IC_50_ values of 16.6 and 1.2 ppm, respectively, without inhibiting fungal growth. Precocene II also inhibited deoxynivalenol production by the fungus in a solid culture on rice with an IC_50_ value of 2.0 ppm.

*O. vulgare* essential oil had as main constituents linalool (45.22%), 4-terpineol (44.11%), thymol (13.40%), α-terpineol (5.96%), and γ-terpinene (5.02%). The literature reports that the carvacrol and thymol are the main constituents and also responsible for antimicrobial activity of essential oil of oregano (Lambert et al., 2001; Chi et al., 2006), however these authors also observed that the level of each component varied in relation to time of year.

The findings of the present study may draw the attention of food industries to conduct further experiments regarding large scale exploitation of essential oils as preservative of food commodities.

## Conclusion

The results showed that the essential oil of *A. conyzoides* inhibits the production of aflatoxin in soybeans more than the essential oil of *O. vulgare*. On the other hand, in the corn samples, the essential oil of *O. vulgare* was more active, so the choice of treatment should take into account the type of substrate. Nowadays, it has been difficult to control exposure of man and animals to mycotoxins, because these compounds naturally occur in the environment.

Thus, the use of natural compounds like essential oils with lower toxicity than that of synthetic products can be a good alternative for aflatoxin control, and the essential oils of *A. conyzoides* and *O. vulgare* may be a possibility for the protection against the production of aflatoxin B1 in corn and soybeans.

### Conflict of interest statement

The authors declare that the research was conducted in the absence of any commercial or financial relationships that could be construed as a potential conflict of interest.
